# PRMT5 silencing selectively affects *MTAP*‐deleted mesothelioma: In vitro evidence of a novel promising approach

**DOI:** 10.1111/jcmm.15213

**Published:** 2020-04-17

**Authors:** Marcella Barbarino, Daniele Cesari, Maria Bottaro, Luca Luzzi, Asadoor Namagerdi, Franca Maria Bertolino, Cristiana Bellan, Fabrizio Proietti, Pasquale Somma, Mariacarolina Micheli, Maria Margherita de Santi, Raffaella Guazzo, Luciano Mutti, Luigi Pirtoli, Piero Paladini, Paola Indovina, Antonio Giordano

**Affiliations:** ^1^ Department of Medical Biotechnologies University of Siena Siena Italy; ^2^ Sbarro Institute for Cancer Research and Molecular Medicine Center for Biotechnology College of Science and Technology Temple University Philadelphia Pennsylvania; ^3^ Department of Medicine, Surgery and Neurosciences Siena University Hospital Siena Italy; ^4^ Agilent Technologies Milan Italy; ^5^ Anatomy and Pathology Unit Ospedale dei Colli AORN, "Monaldi" Naples Italy

**Keywords:** E2F1, epithelial‐to‐mesenchymal transition, Mesothelioma, MTAP, PRMT5

## Abstract

Malignant mesothelioma (MM) is an aggressive asbestos‐related cancer of the serous membranes. Despite intensive treatment regimens, MM is still a fatal disease, mainly due to the intrinsic resistance to current therapies and the lack of predictive markers and new valuable molecular targets. Protein arginine methyltransferase 5 (PRMT5) inhibition has recently emerged as a potential therapy against methylthioadenosine phosphorylase (MTAP)‐deficient cancers, in which the accumulation of the substrate 5'‐methylthioadenosine (MTA) inhibits PRMT5 activity, thus sensitizing the cells to further PRMT5 inhibition. Considering that the *MTAP* gene is frequently codeleted with the adjacent cyclin‐dependent kinase inhibitor 2A (*CDKN2A*) locus in MM, we assessed whether PRMT5 could represent a therapeutic target also for this cancer type. We evaluated PRMT5 expression, the MTAP status and MTA content in normal mesothelial and MM cell lines. We found that both administration of exogenous MTA and stable *PRMT5* knock‐down, by short hairpin RNAs (shRNAs), selectively reduced the growth of *MTAP*‐deleted MM cells. We also observed that *PRMT5* knock‐down in *MTAP*‐deficient MM cells reduced the expression of E2F1 target genes involved in cell cycle progression and of factors implicated in epithelial‐to‐mesenchymal transition. Therefore, PRMT5 targeting could represent a promising new therapeutic strategy against *MTAP*‐deleted MMs.

## 1 INTRODUCTION

Malignant mesothelioma (MM) is a very aggressive tumour of the serous membranes, with the most frequent type developing in the pleura, the membrane covering the lungs and lining the chest cavity. MM is mainly associated with asbestos exposure and is the most common type of asbestos‐related cancer.^1^, ^2^

Despite current intensive treatment regimens, including chemotherapy, radiation and surgery, MM patients’ overall survival (OS) range from 8 to 36 months, depending on the stage and the histological subtype.^3^, ^4^ Many efforts have been made in the past years to obtain better results in terms of survival, but with limited results.^5^, ^6^, ^7^, ^8^ Only recently, in a phase 3 trial (NCT00651456), the addition of bevacizumab to pemetrexed plus cisplatin achieved an improvement of OS.^9^

Therefore, a better understanding of the molecular machinery underlying MM development is required to move forward against this malignancy. In particular, the discovery of crucial pathways for MM cell proliferation and spreading and the identification of new druggable targets and predictive markers for the stratification of patients who could benefit from a specific therapy are fundamental goals of pre‐clinical research aimed to the improvement of patients’ outcome.

Among the most common genetic alterations or deregulated pathways identified in MM, deletions in the cyclin‐dependent kinase inhibitor 2A (*CDKN2A*) locus, inactivation of the retinoblastoma (RB) pathway, mutations in the *BRCA1*‐associated protein 1 (*BAP1*) and neurofibromatosis type 2 (*NF2*) genes, and aberrant regulation of phosphatidylinositol‐4,5‐ bisphosphate 3‐kinase (PI3K)/AKT pathway are all related to MM uncontrolled growth and resistance to treatment‐induced cell death.^10^, ^11^, ^12^ Moreover, MM poor prognosis has also been found to be related to the activation of the epithelial‐to‐mesenchymal transition (EMT) programme^13^, ^14^ a process involving genetic, epigenetic and morphological changes in epithelial cells, leading to acquisition of a fibroblast‐like cell morphology, reduction in cell adhesion and gain of cell motility, which promotes migration and invasion.^15^, ^16^

Deletion on the short arm of chromosome 9, including the *CDKN2A* locus, is one of the first and most common mutations described in MM.^17^ The discovery that *CDKN2A* deletion in cancer cells commonly involves codeletion of adjacent genes opened new perspectives in cancer research with a possible impact also for MM^18^ It has indeed observed that the methylthioadenosine phosphorylase (*MTAP*) gene, encoding a key enzyme in the adenosine and methionine salvage pathway from the substrate 5'‐methylthioadenosine (MTA), is frequently codeleted with *CDKN2A* in different cancer types^19^ including MM^20^, ^21^ The *MTAP* gene has been suggested to be a tumour suppressor, the loss of which results in a higher cell invasive potential and poor prognosis for patients with different cancer types.^22^ Importantly, *MTAP* loss determines the accumulation of the MTA substrate, a natural inhibitor of protein arginine methyltransferase 5 (PRMT5), thus generating a hypomorphic PRMT5 state in MTAP‐deficient cancers that are, in this way, selectively sensitized to further PRMT5 inhibition. This vulnerability can be exploited therapeutically, and PRMT5 targeting in MTAP‐deficient cancers has indeed become the focus of recent research.^23^, ^24^, ^25^

PRMT5 belongs to a family of ten protein arginine methyltransferases (PRMTs) ubiquitously expressed in mammalian cells, which methylate arginine residues on histones and other proteins, although their biological role is still underexplored. PRMT5 regulates a broad range of physiological and cancer‐associated processes, such as DNA damage response, apoptosis control, EMT and inflammation, and is involved in the inhibition of tumour suppressors, including RB proteins, p53, programmed cell death 4 (PDCD4) and activation of survival pathways such as PI3K/AKT axis^26^, ^27^, ^28^, ^29^

Overall, these considerations prompted us to investigate whether PRMT5 could be a valuable MM therapeutic target, the inhibition of which could impact on pathways fundamental for MM biology.

## 2 MATERIALS AND METHODS

### 2.1 Immunohistochemical analysis

Formalin‐fixed, paraffin‐embedded tumour specimens were used for tissue microarray (TMA) construction. Multi‐tissue pleural mesothelioma arrays were obtained from the Section of Pathology, Siena Hospital, Siena, Italy, and the Anatomy and Pathology Unit, Ospedale dei Colli, AORN, ‘Monaldi’, Naples, Italy, and consisted of 2‐mm representative areas of resected tumour and normal pleura controls. From each tissue microarray, 4‐μm‐thick paraffin sections were prepared for immunohistochemistry. Clinical information about mesothelioma specimens is summarized in Table S1.

Based on the expression patterns identified in the resection specimens, the tumour cell staining in TMA was evaluated in comparison with normal pleura. Two pathologists blinded to the clinical data evaluated the staining of each specimen. To avoid inter‐observer variability, the mean value of the scores was adapted for further analysis. The primary rabbit polyclonal anti‐PRMT5 antibody (Abcam, Cambridge, UK, Cat #ab109451, RRID:AB_10863428) at 1:70 dilutions was used according to the manufacturer's instructions.

The assessment of PRMT5 expression levels included the staining intensity and the percentage of stained cells. PRMT5 was analysed for both nuclear and cytoplasmic staining. The staining intensity was scored as 0 = no staining, 1 = moderate expression and 2 = strong expression; the results were categorized according to the following distribution: 0 =< 10%, 1 = 10% – 50% and 2 ≥ 50% staining. The PRMT5 expression score was determined as a combined score of staining intensity and distribution. Samples with a final immunoscore ≥ 2 were considered as PRMT5‐positive.

### 2.2 Cell lines and culture conditions

NCI‐H2452 (Cat# CRL‐5946, RRID:CVCL_1553) and MeT‐5A (Cat# CRL‐9444, RRID:CVCL_3749) cell lines were purchased from the American Type Culture Collection (ATCC, Manassas, Virginia, USA); LP‐9 cells were from Coriell Institute (Camden, New Jersey, USA, Cat# AG07086, RRID:CVCL_E109); IST‐Mes1 (Cat# HTL01005, RRID:CVCL_1311), IST‐Mes2 (Cat# HTL01007, RRID:CVCL_1312) and MPP 89 (Cat# HTL00012, RRID:CVCL_1427) were purchased from the ISTGE Cell Repository (Genoa, Italy); and MMB‐1 (RRID:CVCL_IW98) and REN (RRID:CVCL_M202) were a kind gift of Prof. Giovanni Gaudino (University of Hawaii Cancer Center, Honolulu, Hawaii, USA). All the cell lines were cultured according to the manufacturer's protocols. Human mesothelial cells (HMC‐NEO) immortalized with a PSV3NEO plasmid were kindly provided by Prof. Paolo Pinton (University of Ferrara, Ferrara, Italy).

MMP1, MMP2 and MMP4 mesothelioma cell lines were isolated from patients’ who underwent surgery at the Thoracic Surgery Unit (Siena, Italy) for decortication, without prior chemotherapy or radiotherapy. MMP6 cell line was derived from pleural effusion. All specimens were collected from patients diagnosed for pleural mesothelioma (MMP1, MMP4 and MMP6: epithelioid; MMP2: biphasic) selected for surgery based on the pre‐operative staging and with their written consent. Non‐immortalized HMC1 cells were obtained from pleural effusion of a patient with heart failure. Human investigations were performed after Research Ethics Committee (Comitato Etico Regione Toscana‐Area Vasta Sud Est) approval (#CCMESOLUNG). The study is conformed to the standards of the Declaration of Helsinki. The original pathologic materials were analysed by light microscopic analysis, followed by extensive immunocytochemical analysis using a battery of markers (Table S2).

Both solid tumours and pleural effusions were transported to the laboratory for primary cell culturing within 30 minutes of collection. Solid tissue was minced into small pieces, 1–3 mm, and then incubated in complete medium supplemented with collagenase type I from Clostridium histolyticum (Thermo Fisher Scientific, Cat #17100017) at 200 U/mL concentration for 1 hour to digest collagen and release tumour cells. Macrophages, red blood cells and lymphocytes were the main contaminants; to avoid their interference in the analysis, all the primary cells were used after the 6th passage.

Effusions were centrifuged at 400  *g* for 5 minutes and placed into culture flasks.

All primary cell lines were cultured in Medium 199 (Euroclone, Pero, Cat #ECB2056L), supplemented with 2 mmol/L l‐glutamine (Euroclone, Cat #ECB3000D), 100 U/mL penicillin, 100 μg/mL streptomycin (Euroclone, Cat# ECB3001D), 10% FBS (Euroclone, Cat #0180L), 20 ng/mL hEGF (Sigma‐Aldrich, Cat #H0888), 0.4 µg/ml hydrocortisone (Sigma‐Aldrich, Cat #E9644) at 37°C and 5% CO_2_. All cell lines were routinely passaged every 1–2 weeks.

Mesothelial origin of primary cell cultures was assessed by haematoxylin/eosin, calretinin and WT‐1 staining.

### 2.3 Fluorescence in situ hybridization (FISH)

Qualitative detection of *CDKN2A* gene (green signal) deletions and the classical satellite III region of chromosome 9 (CEN9) (red signal) were detected by fluorescence in situ hybridization (FISH), using SPEC *CDKN2A*/CEN 9 Dual Color Probe ZytoLight (ZytoVision GmbH, Bremerhaven, Germany). Cell lines were fixed with 4% paraformaldehyde for 10 minutes at RT, washed with PBS and pre‐treated with heat pre‐treatment solution citric, following the manufacturer's protocol. FISH was performed with a hybridization automation (HYBrite; Abbott Molecular). Probe was placed on the samples, covered with a glass slide and then sealed with rubber cement. After codenaturation at 78°C for 10 minutes, the probe and the target DNA were allowed to hybridize at 37˚C overnight in a humid and dark atmosphere. Next day, the excess of probe was washed in 25× wash buffer A at 37°C. Slides were air‐dried in the darkness and counterstained with 4',6‐diamidino‐2‐phenylindole (DAPI). Analysis was performed using a fluorescent microscope (Leica, Wetzlar, Germany) and Leica LAS v3.8 Software (Leica) at ×630 magnification, equipped with SpectrumGreen™, SpectrumOrange™ filters. For each specimen, at last 100 intact non‐overlapping nuclei with good signals were required for valid scoring.

### 2.4 LC‐MS/MS measurement of MTA intracellular content

The protocol used is based on the method published by Stevens and coworkers^30^ with some modifications. After removing the cell culture medium and washing the cells with PBS buffer, 2 × 10^5^ cells were scraped directly into 500 µL of pure methanol spiked with 2.48 nmol/L of stable isotope‐labelled internal standard. Scraped cells were centrifuged (100 *g*, 5 minutes, room temperature), and the supernatant was collected. The cell pellet was washed twice with 200 µL methanol and centrifuged, and all supernatants were combined, dried and reconstituted in 100 µL of water. All solvents for sample preparation and LC‐MS/MS were HPLC grade and purchased from Sigma‐Aldrich. MTA was obtained from Sigma‐Aldrich (Cat #D5011) and labelled. ^15^N5 8‐hydroxy‐2'‐deoxyguanosine was obtained from Cambridge Isotope Laboratories Inc. The water used was purified by means of a MilliQ (Millipore).

LC‐MS/MS was performed using an Agilent 1200 SL HPLC system and an ABSciex API 4000 triple quadrupole mass spectrometer (AB Sciex), which was equipped with a turbo ion spray source (completely controlled by Analyst version 1.4.2). A Luna C18 (4.6 × 150 mm, 5 µm, 100Å) reversed phase column (Phenomenex) was used. LC separation was carried out using a mobile phase consisting of 0.1% acetic acid in water (Solvent A) and 0.1% acetic acid in acetonitrile (Solvent B). The gradient employed was as follows: 0‐2 minutes isocratic 5% solvent B, 2‐10 minutes linear increase from 5% to 100% solvent B, hold at 100% solvent B for 3 minutes, and 3 minutes post‐run equilibration. The flow rate was set to 500 µL/min. Sample volumes of 20 µL were injected. The API 4000 mass spectrometer was operated in positive mode using turbo ion spray with the following parameters: gas 1 as 55, gas 2 as 45 and the curtain gas as 17 (all arbitrary units).

The turbo ion spray source was heated to 450°C. The declustering potential was set to 47 and the entrance potential to 9.4 V.

The transition analysed was 296.3/136 (MTA Quantifier) 296.3/119.2 (MTA Qualifier) 269.0/173.0 (IS).

### 2.5 Cell treatment with MTA

MTA was obtained from Sigma‐Aldrich (Cat #D5011). Stock solutions of the drug were prepared in dimethyl sulfoxide cell culture grade (DMSO) (Euroclone, Cat #EMR385100) and stored at −20°C.

Cells were seeded in 96‐well plates (Costar/Sigma‐Aldrich, Cat #3599) 24 hours before treatment with MTA and incubated for further 72 hours. Control cells were treated with DMSO at the same amount used to deliver the molecule. Each experiment was conducted in triplicate. Cell viability was evaluated by sulforhodamine B (SRB) assay (Sigma‐Aldrich, Cat #230162), as previously described.^31^ Absorbance values were measured with a microplate reader (Euroclone) at 540 nm. The half maximal inhibitory concentration (IC50) values were calculated using GraphPad Prism 6 (GraphPad Software Inc; http://www.graphpad.com/scientificsoftware/prism/ ).

### 2.6 Generation of cells stably expressing shRNA constructs

NCI‐H2452, IST‐Mes2, Met‐5A, MMB‐1 and MPP 89 cell lines were transduced with lentiviral vectors expressing short hairpins RNAs against *PRMT5* (Sigma‐Aldrich, SHCLNV NM_006109, Mission®) or sham sequences (Sigma‐Aldrich, SHC016V PLKO.1‐PURO non‐target control, Mission®) according to the manufacturer's instructions. Cell lines were selected with puromycin (Sigma‐Aldrich, Cat #P7255), and *PRMT5* knock‐down was verified by real‐time qRT‐PCR and Western blotting.

### 2.7 Real‐time Reverse Transcriptase‐Quantitative PCR (RT‐qPCR)

Total RNA was isolated from cell lines using the RNeasy Mini kit (Qiagen, Hilden, Germany Cat #74106). RNA concentration was determined using a NanoDrop™ ND‐1000 (Thermo Fisher). Complementary DNA (cDNA) was synthesized from 500 ng of RNA using the iScript cDNA Synthesis Kit (Bio‐rad, Hercules, Cat #1708891BUN) and amplified in the LightCycler™ instrument (Roche Applied Sciences) using SsoAdvanced™ Universal SYBR® Green Supermix (Bio‐rad, Cat #1725274) according to the manufacturer's instruction. The primers used were from Bio‐Rad: *MTAP*, Assay ID qHsaCED0004212; *PRMT5*, Assay ID qHsaCED0042792; *E2F1*, Assay ID qHsaCED0042864; *TS*, Assay ID qHsaCED0047209; *RBL2*, Assay ID qHsaCED0042975; *CCNA2*, Assay ID qHsaCID0017452; *CCNE*, Assay ID qHsaCID0015131; *EZH2*, Assay ID qHsaCED0043150; *MMP9*, Assay ID qHsaCID0011597; *GAPDH* and Assay ID qHsaCED0038674. The housekeeping glyceraldehyde‐3‐phosphate dehydrogenase (*GAPDH*) gene was used to normalize the expression of genes of interest. Gene expression levels were calculated by the 2^−ΔΔ^ *^C^* ^t^ method.^32^

### 2.8 Protein extraction and Western Blotting

For Western blotting analysis, cells were harvested on ice and lysed as previously described^16^ Equal amounts of proteins (30 µg) per sample were electrophoresed and, after transferring to nitrocellulose membranes (Bio‐Rad), were incubated overnight at 4°C with the following antibodies: (Abcam, Cat #ab96231, RRID:AB_10677616), PRMT5 (Abcam, Cat #ab109451, RRID:AB_10863428), N‐cadherin (Santa Cruz Biotechnology, Cat #sc‐31030, RRID:AB_2077520), E‐cadherin (Santa Cruz Biotechnology, Cat #sc‐7870, RRID:AB_2076666), smooth muscle actin (BioGenex, Cat #MU128‐UC, RRID:AB_2335623), RB (Santa Cruz Biotechnology, Cat #sc‐50, RRID:AB_632339), phospho‐RB (Cell Signaling Technology, Cat #3590, RRID:AB_2177182), β‐actin (Sigma‐Aldrich, Cat #A5316, RRID:AB_476743) and GAPDH (Santa Cruz, Cat #sc‐2577, RRID:AB_10167668).

Membranes were washed with TBS with 0.1% Tween‐20 and incubated with horseradish peroxidase‐conjugated secondary antibodies for 1 hour at room temperature. Membranes were washed before chemiluminescence detection using Clarity ECL reagents (Bio‐Rad, Cat #1705061).

### 2.9 Cell growth curves

Single‐cell suspension cultures of log‐phase growing parental/transduced NCI‐H2452, IST‐Mes2, Met‐5A, MMB‐1 and MPP 89 cells were seeded in triplicate in Petri dishes (35 mm diameter). After 24, 48 and 72 hours, the cells from each well were harvested by trypsinization and viable cells were counted in a haemocytometer chamber. Cell viability was evaluated by the trypan blue dye exclusion test.

### 2.10 Statistical analysis

Statistical analyses were performed using the GraphPad Prism Software, version 5.01 for Windows. Statistically significant differences between the means of multiple matched groups were evaluated by one‐way repeated measures ANOVA with Tukey's post‐test. To compare the means of 2 unmatched groups, we used the two‐sided unpaired Student's *t* test. Statistically significant differences among the growth curves were evaluated by two‐way repeated measures ANOVA *P* < .05 was considered statistically significant.

## 3 RESULTS

### 3.1 PRMT5 expression in MM and normal mesothelial cells

**Figure 1:**
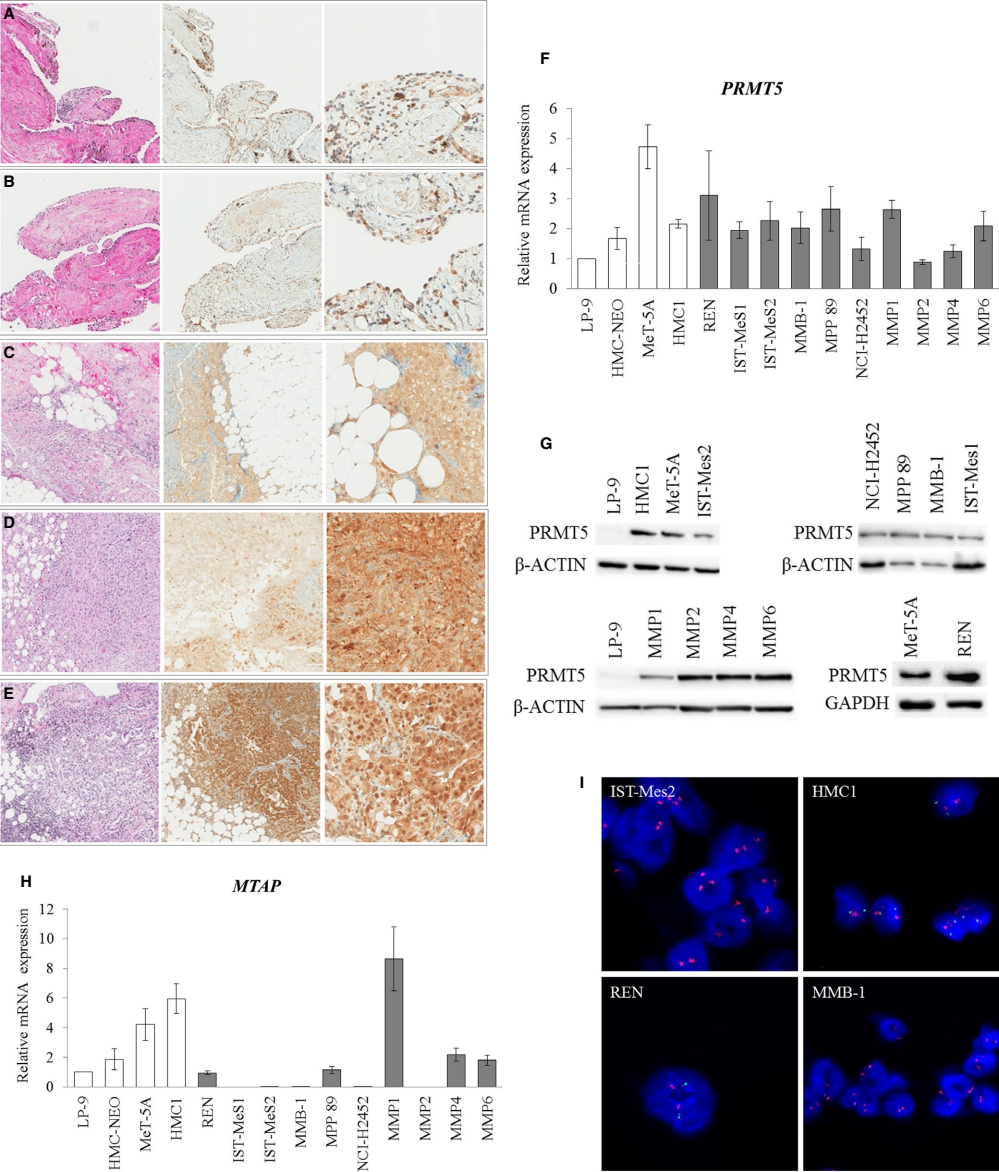
PRMT5 and MTAP expression in MM and normal mesothelial cells. A‐E, PRMT5 expression and localization were analysed in formalin‐fixed, paraffin‐embedded tissues from normal pleural (n = 10) and MM samples (n = 58). From left to right: haematoxylin and eosin staining (magnification 10×), PRMT5 immunostaining (magnification 10×) and PRMT5 immunostaining at a higher magnification (40×). Normal pleural tissues (A, B) show a weak expression of PRMT5, whereas in MM specimens PRMT5 is expressed at high levels in both nuclei and cytoplasms (D, E). In Figure 1C, A representative MM case with cytoplasmic localization of PRMT5 is shown. F, Real‐time qRT‐PCR analysis of *PRMT5* expression in normal mesothelial (white bars of the histogram) and MM (grey bars) cell lines. *PRMT5* expression was calculated by the 2^−ΔΔ^ *^C^* ^t^ method relatively to the normal mesothelial cells, LP‐9, used as a calibrator. Data are reported as means ± SD of three independent experiments. (G) Western blotting analysis of PRMT5 expression in a panel of normal mesothelial and MM cell lines. Antibodies against GAPDH and β‐actin were used as loading controls. H, Real‐time qRT‐PCR analysis of *MTAP* expression in normal mesothelial (white bars of the histogram) and MM (grey bars) cell lines. *MTAP* expression was calculated by the 2^−ΔΔ^ *^C^* ^t^ method relatively to the normal mesothelial cells, LP‐9, used as a calibrator. Data are reported as means ± standard deviations of three independent experiments. IST‐Mes1, IST‐Mes2, MMB‐1, NCI‐H2452 and MMP2 cell lines were *MTAP*‐negative. All the remaining cell lines showed different levels of *MTAP* transcript. I, Two‐colour FISH labelling with the *CDKN2A* probe (green) and the chromosome 9 centromere (CEN9) probe (red), showing representative *MTAP‐*intact cells (HMC1 and REN), with a normal copy number of *CDKN2A* (two copies for each probe), and representative *MTAP*‐deficient cell lines (IST‐Mes2 and MMB‐1) with the homozygous deletion of *CDKN2A*. Homozygous deletion is defined as the lack of both green *CDKNA2* signals (9p21) in the presence of at least one centromere of chromosome 9 signal. Nuclei were counterstained with DAPI

To explore the role of PRMT5 in MM, we first analysed its expression in 58 MM tissue specimens and 10 normal pleural samples through immunohistochemistry. Our analysis revealed that 43% of the MM samples expressed PRMT5 in both nuclei and cytoplasms (Figure 1, D and E), 19% and 31% showed a cytoplasmic (Figure 1C) or nuclear PRMT5 localization, respectively, and only 7% did not express PRMT5. All the normal pleura samples expressed PRMT5, mainly in both nuclei and cytoplasms, although at a lower level with respect to cancer cells (Figure 1A,B).

Then, we analysed PRMT5 expression in a panel of previously established/commercial (REN, IST‐Mes1, IST‐Mes2, MMB‐1, MPP 89 and NCI‐H2452) and patient‐derived primary (MMP1, MMP2, MMP4 and MMP6) MM cell lines and in normal mesothelial cells (LP‐9, HMC‐NEO, MeT‐5A and HMC1), by both real‐time quantitative reverse transcription‐PCR (qRT‐PCR) and Western blotting, and found that PRMT5 was expressed, although at different levels, in all the cells analysed (Figure 1F,G).

### 3.2 Exogenous MTA administration reduces the growth of *MTAP*‐deleted MM cell lines

Before assessing whether MTA accumulation in *MTAP*‐deleted MM cells renders, indeed, these cells more susceptible to further PRMT5 inhibition, we analysed *MTAP* expression in the panel of MM and normal mesothelial cell lines by real‐time qRT‐PCR. We observed that *MTAP* was expressed, although at different levels, in all normal cells and in REN, MPP 89, MMP1, MMP4 and MMP6 cells, whereas its transcript was undetectable in IST‐Mes1, IST‐Mes2, MMB‐1, NCI‐H2452 and MPP2 cells (Figure 1H). For NCI‐H2452, IST‐Mes1 and MPP 89, the results were as expected, based on the *MTAP* status previously reported by the Sanger Institute, Catalogue of Somatic Mutations in Cancer (http://cancer.sanger.ac.uk/cancergenome/projects/cell_lines/). Consistent results were also obtained through Western blotting (Figure S1) and fluorescence in situ hybridization (FISH) analysis for selected cell lines (Figure 1I and Figure S2). MTAP‐deficient and MTAP‐proficient cell lines are summarized in Table 1.

**Table 1:** *MTAP* status in normal mesothelial cell lines, in MM cell lines and in MM primary cultures

Cell line	*MTAP* status
LP‐9	+
HMC‐NEO	+
MeT‐5A	+
HMC1	+
REN	+
IST‐Mes1*	−
IST‐Mes2	−
MMB‐1	−
MPP 89*	+
NCI‐H2452*	−
MMP1	+
MMP2	−
MMP4	+
MMP6	+

Note

The asterisks indicate the *MTAP* status that has previously been reported (Sanger Institute, Catalogue of Somatic Mutations in Cancer, (http://cancer.sanger.ac.uk/cancergenome/projects/cell_lines/).

**Figure 2:**
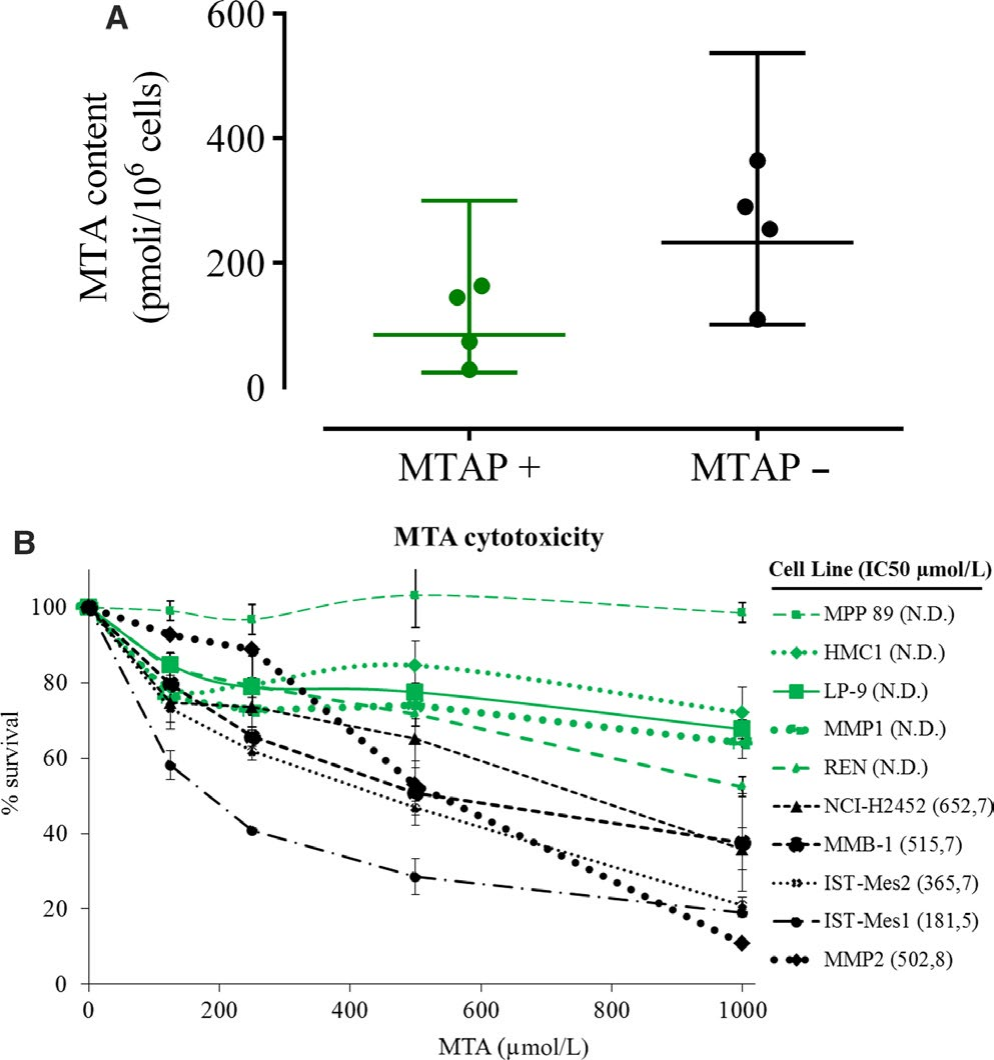
*MTAP‐*deleted cells accumulate MTA and are susceptible to its further addition. A, Intracellular MTA levels were measured by high‐performance liquid chromatography‐tandem mass spectrometry (LC‐MS/MS) in a panel of 8 cell lines including 4 *MTAP*‐deficient MM cell lines (IST‐Mes1, IST‐Mes2, MMB‐1 and NCI‐H2452) and 4 *MTAP*‐proficient normal mesothelial (LP‐9 and HMC‐NEO) and MM (REN and MPP 89) cell lines. The dot plot reports the MTA levels both individually for each cell line (symbols) and as mean values (lines) with standard deviation calculated on all cells for each group. Each data point represents the mean of at least two independent measures for each cell line. The means with standard deviations of MTA levels for each cell line are reported in Table S3. Statistical analysis was performed by two‐sided unpaired Student's t test, and significance was indicated with *: (*P* < .05). On average, *MTAP*‐deficient cells have approximately 2.5 times the intracellular MTA level of *MTAP*‐intact cells (mean MTA level in *MTAP*‐deficient cells = 102.5 pmol/10^6^ cells; mean MTA level in *MTAP*‐intact cells = 254.4 pmol/10^6^ cells). B, Seventy‐two hours IC50 values of MTA for the *MTAP*‐deleted cells, as determined through SRB assay; MTA IC50 values were not determinable (ND) for *MTAP*‐positive cells under our experimental conditions. Cell viability data were expressed as percentage with respect to the control cells treated with dimethyl sulfoxide (DMSO) alone; the means and standard deviations of three experiments are shown

Then, we analysed the intracellular MTA content in selected *MTAP*‐positive normal mesothelial and MM cell lines and in *MTAP*‐negative MM cells by high‐performance liquid chromatography‐tandem mass spectrometry (LC‐MS/MS). Consistent with what previously demonstrated in other cancer types,^24^, ^25^, ^33^, ^34^ *MTAP*‐negative MM cells showed a significantly higher mean intracellular MTA level compared with that of *MTAP*‐positive cells (Figure 2A and Table S3). We then verified whether the exogenous addition of MTA could impact on the cell viability of *MTAP‐*deleted cells, without affecting *MTAP*‐positive cells. To this purpose, we treated MM and mesothelial cell lines with increasing MTA concentrations and, after 72 hours, we evaluated cell viability by sulforhodamine B (SRB) assay. We observed that MTA had cytotoxic effects on all *MTAP‐*deleted cells, resulting in the indicated half maximal inhibitory concentration (IC50) values (Figure 2B); conversely, MTA IC50 values were not determinable (ND) for *MTAP*‐positive cells under our experimental conditions. These results support the hypothesis that *MTAP*‐deleted MM cells could be selectively targeted for their susceptibility to PRMT5 inhibition due to the accumulation of its natural inhibitor MTA.

### 3.3 *PRMT5* knock‐down decreases proliferation of MTAP‐negative MM cells

**Figure 3:**
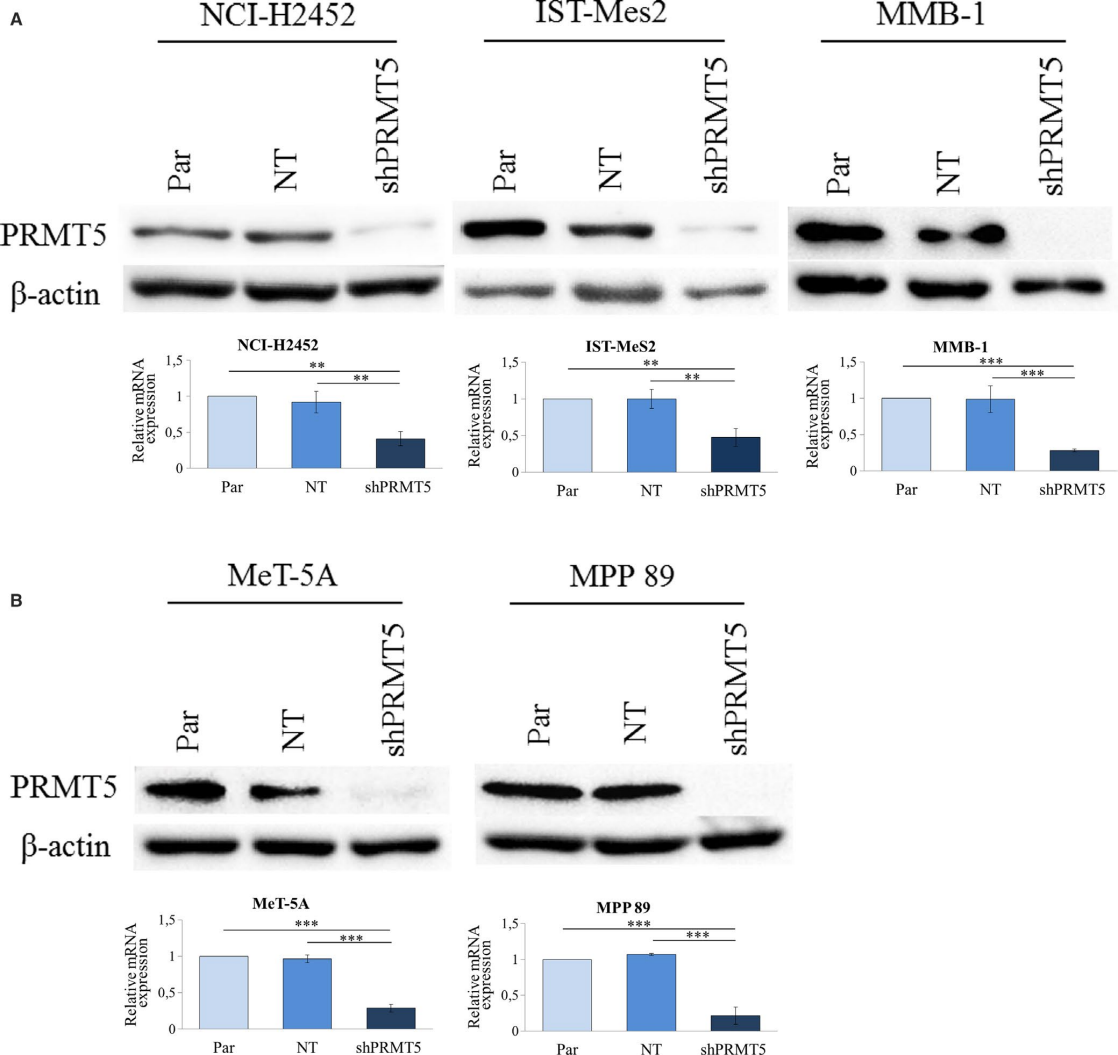
*PRMT5* silencing by shRNA in *MTAP*‐negative (NCI‐H2452, MMB‐1 and IST‐MeS2) and *MTAP*‐positive (MPP 89) MM cells and in a non‐tumour mesothelial cell line (MeT‐5A). A, Western blotting analysis of PRMT5 in cells expressing shRNAs against *PRMT5* (shPRMT5), in cells expressing a non‐targeting (NT) control shRNA and in non‐transduced parental cells (Par). An antibody against β‐actin was used as a loading control. B, Real‐time qRT‐PCR analysis of *PRMT5* in the same cells as in (A). *PRMT5* expression was calculated by the 2^−ΔΔ^ *^C^* ^t^ method in shPRMT5 and NT‐shRNA expressing cells relatively to parental cells. Results are reported as means with standard deviations of three independent experiments. Statistical analysis was performed by subjecting the Δ*C*_t_ values to one‐way repeated measures ANOVA with Tukey's post‐test. Statistically significant differences are indicated with: **very significant (*P* < .01) and ***: extremely significant (*P* < .001)

To verify whether PRMT5 inhibition could indeed selectively affect *MTAP*‐deficient MM cell growth, we stably transduced with lentiviral vectors expressing shRNAs against *PRMT5* (shPRMT5) or a non‐targeting (NT) control shRNA, the *MTAP‐*negative MM cells, NCI‐H2452, MMB‐1 and IST‐Mes2, the *MTAP‐*positive MM cells, MPP 89, and the non‐tumour mesothelial cells, MeT‐5A. Infected cells were selected for puromycin resistance, and *PRMT5* silencing was verified by Western blotting (Figure 3A) and real‐time qRT‐PCR (Figure 3B).

**Figure 4:**
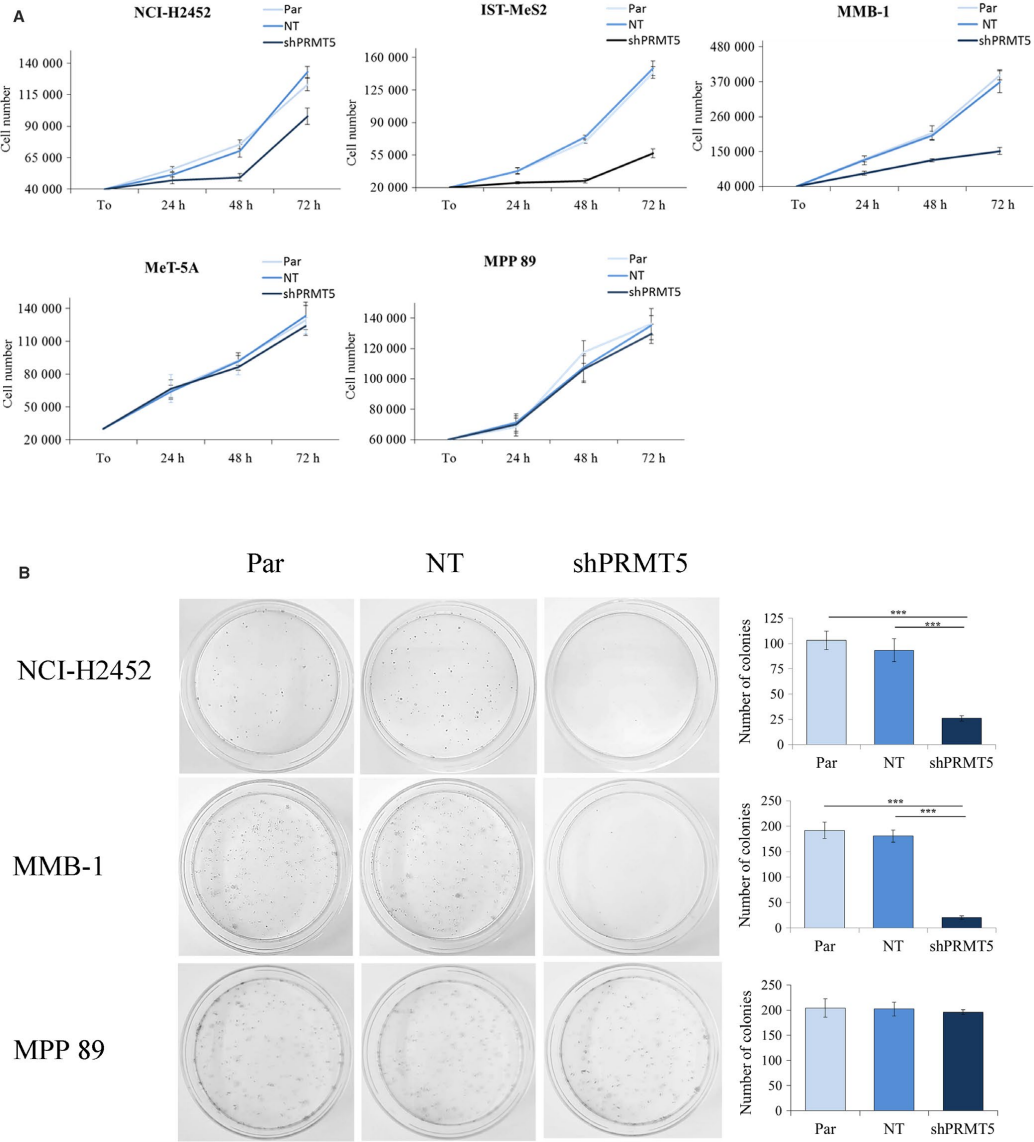
*PRMT5* silencing selectively affects the growth and clonogenic potential of *MTAP*‐deleted MM cells. A, Growth curves of non‐transduced parental cells (Par) and of cells expressing shRNAs against *PRMT5* (shPRMT5) or a non‐targeting (NT) control shRNA. Cells were seeded at time‐point zero (T0) and counted at 24, 48 and 72 h after seeding. Results are reported as means with standard deviations of three independent experiments. *PRMT5* knock‐down significantly impaired the growth of the *MTAP*‐negative MM cells, NCI‐H2452, IST‐MeS2 and MMB‐1 (statistical significance was determined by two‐way repeated measures ANOVA, *P* < .001), without affecting the growth of the *MTAP*‐positive MM cells, MPP 89, and non‐tumour mesothelial cells, Met‐5A. B, Representative dishes, out of three clonogenic assays, of parental MM cells and of MM cells expressing shPRMT5 and NT‐shRNA. The mean numbers of colonies with standard deviations obtained from the three experiments are reported in the histograms. Data were subjected to one‐way repeated measures ANOVA with Tukey's post‐test. *PRMT5* silencing significantly affected the colony formation ability of NCI‐H2452 and MMB‐1 (***P < .001), without impairing the clonogenic potential of MPP 89 cells

In the *MTAP*‐deleted MM cells, NCI‐H2452, MMB‐1 and IST‐Mes2, *PRMT5* silencing significantly inhibited cell growth, compared to parental and NT‐shRNA expressing control cells (Figure 4A). Conversely, in the *MTAP*‐positive MM cells, MPP 89, and non‐tumour mesothelial cells, MeT‐5A, *PRMT5* silencing had no effect.

We then analysed the impact of *PRMT5* silencing on the clonogenic potential of selected MM cell lines, representative of *MTAP*‐positive (MPP 89) and *MTAP*‐negative (NCI‐H2452, MMB‐1) cells. As shown in Figure 4B, colony formation was significantly impaired by *PRMT5* silencing only in *MTAP*‐negative MM cells.

### 3.4 *PRMT5* knock‐down reduces the expression of E2F1 target genes involved in cell cycle progression in MTAP‐negative MM cells

**Figure 5:**
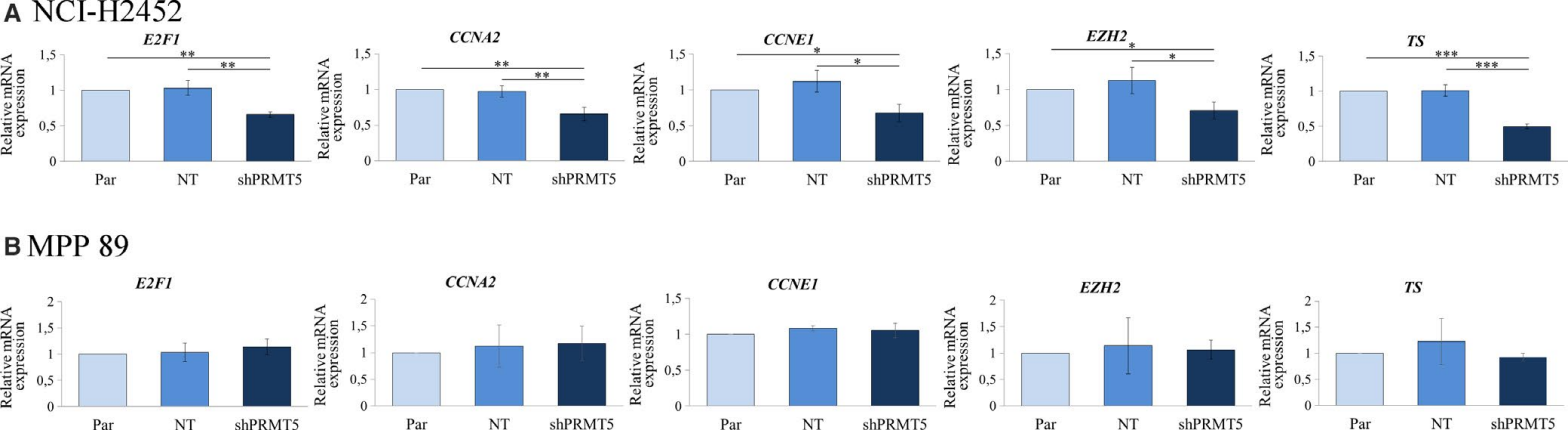
Effect of PRMT5 silencing on the E2F1 pathway in MM cells. A, The expression of *E2F1* and of the E2F1‐regulated genes, cyclin A2 (*CCNA2*), cyclin E1 (*CCNE1*), enhancer of zeste homolog 2 (*EZH2*) and thymidylate synthase (*TS*), was evaluated by real‐time qRT‐PCR analysis in NCI‐H2452 cells expressing shPRMT5 or the NT control, relatively to non‐transduced parental cells. Relative expression was calculated by the 2^−ΔΔ^ *^C^* ^t^ method. Results are reported as means with standard deviations of three independent experiments. Statistical analysis was performed by subjecting the Δ*C*_t_ values to one‐way repeated measures ANOVA with Tukey's post‐test. Statistically significant differences are indicated with: *significant (*P* < .05), **very significant (*P* < .01) and ***: extremely significant (*P* < .001). B, Relative expression of the same E2F1‐regulated genes was also evaluated in the *MTAP*‐positive cells, MPP 89. No statistically significant differences were observed among parental cells, NT‐shRNA expressing cells and shPRMT5 expressing cells

To explore at the molecular level the proliferative role of PRMT5 in *MTAP*‐deleted MM cells, we analysed the effect of PRMT5 knock‐down on the expression of genes involved in cancer cell growth. Among the broad range of factors regulated by PRMT5, we first investigated whether *PRMT5* silencing could affect the E2F pathway in *MTAP*‐deleted MM cells. We observed that *PRMT5* knock‐down inhibited the E2F1 growth‐promoting signalling, as suggested by the down‐regulation of the E2F1‐regulated genes ^35^, ^36^, ^37^, ^38^, ^39^ cyclin A2 (*CCNA2*), cyclin E1 (*CCNE1*), enhancer of zeste homolog 2 (*EZH2*), thymidylate synthase (*TS*) and *E2F1* itself (Figure 5A). We analysed the expression of the same E2F1‐regulated genes also in the *MTAP*‐positive, *PRMT5*‐silenced cells, MPP 89. Consistent with our observation that MPP 89 cell growth is unaffected by PRMT5 inhibition (Figure 4A), we did not found changes in *E2F1*, *EZH2*, *CCNA2*, *CCNE1* and *TS* expression (Figure 5B), supporting a critical role of the E2F1‐transcriptional activity in PRMT5‐mediated proliferation specifically in *MTAP*‐deleted MM cells.

### 3.5 PRMT5 knock‐down impacts on the expression of epithelial‐to‐mesenchymal transition markers in *MTAP*‐negative MM cells

EMT is a cellular process whereby epithelial cells lose some of their typical characteristics and acquire mesenchymal properties.^40^ This phenomenon has a key role in cancer motility and drug resistance and has recently been demonstrated to be promoted by PRMT5.^41^

**Figure 6:**
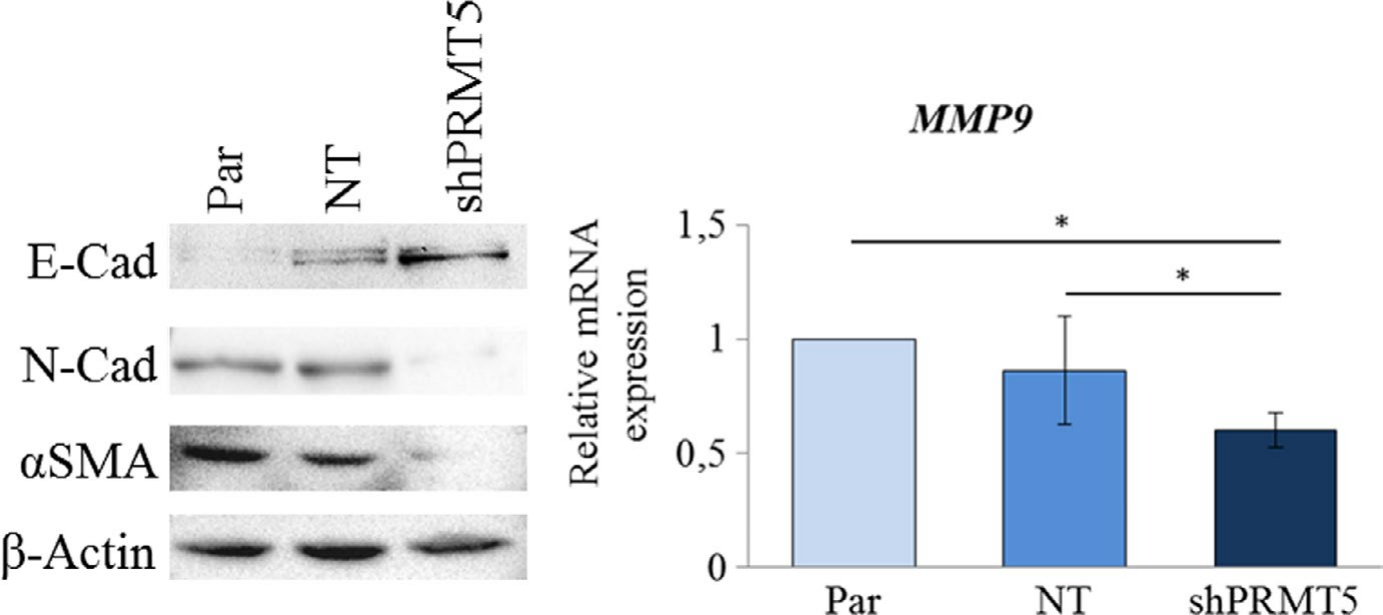
Effect of *PRMT5* silencing on EMT marker expression in NCI‐H2452 *MTAP*‐negative MM cells. Western blotting analysis of E‐cadherin, N‐cadherin and α‐smooth muscle actin (αSMA) in parental (Par) NCI‐H2452 cells and in these cells expressing a non‐targeting (NT) shRNA or shRNA against *PRMT5* (shPRMT5). A β‐actin antibody was used as a loading control. The same cells were analysed for matrix metallopeptidase 9 *(MMP9)* expression by real‐time qRT‐PCR *MMP9* expression was calculated by the 2^−ΔΔ^ *^C^* ^t^ method relatively to parental cells. Results are reported as means with standard deviations of two independent experiments. Statistical analysis was performed by subjecting the Δ*C*_t_ values to one‐way repeated measures ANOVA with Tukey's post‐test. Statistically significance is indicated with: *significant (*P* < .05)

Therefore, we investigated the effect of *PRMT5* knock‐down in the *MTAP*‐negative cells, NCI‐H2452, on the expression of EMT markers. As shown in Figure 6, *PRMT5* depletion led to the up‐regulation of the epithelial marker, E‐cadherin, and the concomitant down‐regulation of the mesenchymal markers, N‐cadherin, *α*‐smooth muscle actin (*α‐*SMA) and matrix metallopeptidase 9 (*MMP9*).

## 4 DISCUSSION

Recent advances in cancer metabolism research have identified molecular defects leading to vulnerabilities, which can be exploited therapeutically^42^ These molecular alterations can also represent biomarkers helping move towards personalized medicine. Among these defects, an altered expression of metabolic enzymes is frequent in cancer and can also occur as a passenger event. Indeed, genomic deletions of tumour suppressor genes can involve adjacent metabolic genes, thus generating a targetable vulnerability^42^ PRMT5 dependence is an example of this phenomenon, which has been termed collateral lethality^43^ Indeed, the passenger deletion of *MTAP*, resulting from the deletion of the *CDKN2A* tumour suppressor gene, generates a selective vulnerability to PRMT5 inhibition^24^ In the recent years, interest for PRMT5 as a new druggable target for anticancer therapy has increased exponentially and great efforts have been made to discover new and selective PRMT5 inhibitors^44^ Among a number of chemical compounds demonstrated to be active in pre‐clinical studies, GSK3326595 (NCT02783300), PF‐06939999 (NCT03854227), JNJ‐64619178 (NCT03573310) and PRT543 (NCT03886831) have entered clinical trials.

Although PRMT5 has been proposed as a possible therapeutic target also for MM,^18^ its role has not yet been extensively explored in this malignancy. In the present study, we first analysed the expression of PRMT5 in normal mesothelial and MM cell lines and archival tissue specimens and found that PRMT5 was expressed in both normal and cancer cells, although at a higher level in MM cells. PRMT5 expression has been studied in various cancer types, in which its level and localization appeared to vary depending on the organs of origin and the histological types^41^, ^45^, ^46^, ^47^, ^48^ However, we did not find in MM any correlation between the PRMT5 expression levels or intracellular distribution and the histological grade (data not shown).

We then analysed the *MTAP* status and MTA intracellular content in a panel of MM and normal mesothelial cell lines and, in line with what previously reported for other cancer types,^24^, ^25^ we found a higher MTA content in *MTAP*‐deleted MM cells and a greater cytotoxic effect of exogenous addition of MTA, the natural inhibitor of PRMT5, in these cells. Consistently, shRNA silencing of *PRMT5* led to a decreased cell growth and clonogenic potential selectively in *MTAP*‐deleted MM cells, similar to a previous observation ^49^

To analyse at the molecular level the growth‐promoting activity of PRMT5 in MM cells, we first focused on the E2F pathway. We observed that *PRMT5* knock‐down led to a decreased expression of E2F1 target genes in *MTAP*‐negative cells. E2F1 has previously been found to exert controversial roles in different human cancers, promoting either proliferation or tumour suppression, depending on the context ^50^ Although E2F1 has been proposed as a therapeutic target and it is an independent prognostic factor for many cancers,^51^, ^52^, ^53^, ^54^ the role of E2F1 in MM has poorly been investigated. Among other mechanisms, E2F1 can be regulated by PRMT5 arginine methylation; it has indeed been demonstrated that the binding of cyclin A to E2F1 augments PRMT5 methylation of E2F1, thus ensuring that it is locked in a cell cycle progression mode^55^ Based on the observed down‐regulation of cyclin A, together with the down‐regulation of other E2F1 target genes, in *PRMT5*‐silenced MM cells, it could be hypothesized that the E2F1 activity in MM is affected by PRMT5‐mediated arginine methylation. However, this needs further investigation.

Interestingly, among the E2F1 target genes down‐regulated by *PRMT5* silencing and involved in cell cycle progression, the *EZH2* gene encodes a factor implicated in the epigenetic regulation of gene expression^56^ which is a negative prognostic factor and a potential therapeutic target in MM^57^

Finally, we identified a subset of EMT markers regulated by PRMT5 in MM, including E‐cadherin, N‐cadherin, α‐smooth muscle actin and MMP9, indicating that targeting PRMT5 could hamper this crucial process for cancer progression.

## 5 DISCUSSION

To the best of our knowledge, this is the first study extensively exploring the possible therapeutic potential of targeting PRMT5 in *MTAP*‐deleted MM cells, also showing the effect of its silencing on the expression of genes implicated in MM growth and progression. In agreement with previous studies underlining the potential value of PRMT5 as a therapeutic approach,^58^, ^59^, ^60^, ^61^ our results represent a starting point for the evaluation of PRMT5 inhibitors also in *MTAP*‐deleted mesothelioma.

## CONFLICTS OF INTEREST

The authors have no conflicts of interest to declare.

## AUTHOR CONTRIBUTIONS

MB^1^ designed the research study and wrote the manuscript. MB^1^ and AG contributed to funding acquisition. AG, PI and LM reviewed the manuscript. DC, MB^2^, FMB and AN performed biological and molecular experiments. FP performed LC‐MS/MS measurement of MTA. PI provided suggestions on data presentation and performed statistical analysis. LL, LP and PP furnished patient‐derived biopsies for cell lines establishment. CB, MdS, RG, MM and PS provide IHC analysis and results.

MB^1^: Marcella Barbarino; MB^2^: Maria Bottaro.

## Supporting information

Fig S1

Fig S2

Table S1

Table S2

Table S3

## Data Availability

The data that supports the findings of this study are available in the supplementary material of this article.
