# Supplementary material for: PRMT5 silencing selectively affects MTAP‐deleted mesothelioma: In vitro evidence of a novel promising approach
Source: J Cell Mol Med. 2020 Apr 17;24(10):5565–77. doi: 10.1111/jcmm.15213 (PMC7214180; doi:10.1111/jcmm.15213)
Supplement: Supplementary file 1 — Fig S1 [file JCMM-24-5565-s001.doc]

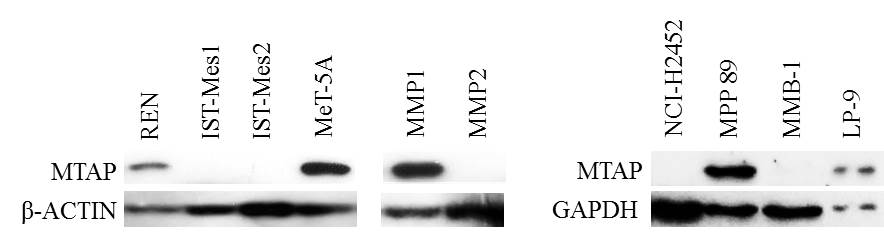


Figure S1. Western blotting analysis of MTAP expression in selected normal mesothelial and selected MM cell lines. Antibodies against GAPDH and β-actin were used as loading controls.
