# Supplementary material for: PRMT5 silencing selectively affects MTAP‐deleted mesothelioma: In vitro evidence of a novel promising approach
Source: J Cell Mol Med. 2020 Apr 17;24(10):5565–77. doi: 10.1111/jcmm.15213 (PMC7214180; doi:10.1111/jcmm.15213)
Supplement: Supplementary file 2 — Fig S2 [file JCMM-24-5565-s002.doc]

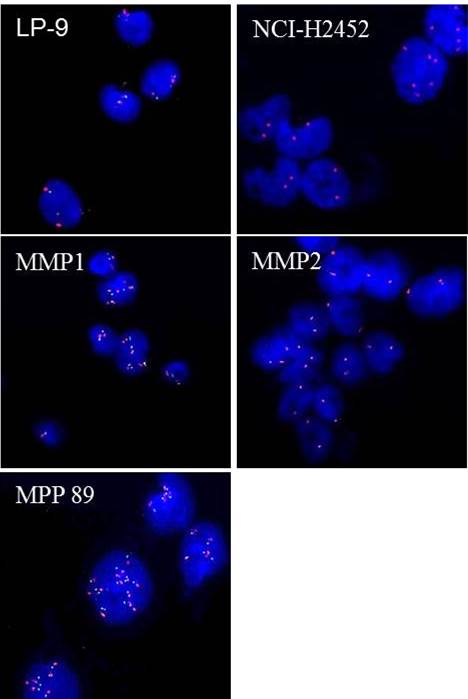


Figure S2. Two-color FISH labeling with the *CDKN2A* probe (green) and the chromosome 9 centromere (CEN9) (red), showing *MTAP-*intact cells (LP-9, MMP1 and MPP 89) with a normal copy number of *CDKN2A* (two copies for each probes) and *MTAP*-deficient cell lines (NCI-H2452 and MMP2) with the homozygous deletion of *CDKN2A*. Nuclei were counterstained with DAPI.
