# Supplementary material for: PRMT5 silencing selectively affects MTAP‐deleted mesothelioma: In vitro evidence of a novel promising approach
Source: J Cell Mol Med. 2020 Apr 17;24(10):5565–77. doi: 10.1111/jcmm.15213 (PMC7214180; doi:10.1111/jcmm.15213)
Supplement: Supplementary file 3 — Table S1 [file JCMM-24-5565-s003.doc]

| Males | 77,5 % |
| --- | --- |
| Females | 22,5 % |
| Epithelioid histotype | 67,5% |
| Biphasic histotype | 20% |
| Sarcomatoid histotype | 12,5% |

Table S1. Clinical data of mesothelioma specimens expressed as percentage of total analyzed samples. All patients have Italian nationality. Asbestos exposure was ascertained in 40% of patients.
