# Supplementary material for: PRMT5 silencing selectively affects MTAP‐deleted mesothelioma: In vitro evidence of a novel promising approach
Source: J Cell Mol Med. 2020 Apr 17;24(10):5565–77. doi: 10.1111/jcmm.15213 (PMC7214180; doi:10.1111/jcmm.15213)
Supplement: Supplementary file 4 — Table S2 [file JCMM-24-5565-s004.doc]

| **Marker** | **Cell Type** | **Staining** |
| --- | --- | --- |
| Calretinin (nuclear and cytoplasmatic) | Mesothelial | Positive |
| WT1 (nuclear) | Mesothelial | Positive |
| CD31 | Endothelial cells | Negative |
| CD34 | Endothelial cells | Negative |
| α-SMA | Cancer associated fibroblast | Negative |

Table S2. Markers used to characterize patients’ derived cell culture according the "Guidelines for Pathologic Diagnosis of Malignant Mesothelioma 2017, Update of the Consensus Statement From the International Mesothelioma Interest Group" (Arch Pathol Lab Med. 2018;142:89–108; doi: 10.5858/ arpa.2017-0124-RA).

(WT1: Wilms' tumor 1; CD31: Cluster of differentiation 31; CD34: Cluster of differentiation 34; α-SMA : Alpha-smooth muscle actin).
