# Supplementary material for: PRMT5 silencing selectively affects MTAP‐deleted mesothelioma: In vitro evidence of a novel promising approach
Source: J Cell Mol Med. 2020 Apr 17;24(10):5565–77. doi: 10.1111/jcmm.15213 (PMC7214180; doi:10.1111/jcmm.15213)
Supplement: Supplementary file 5 — Table S3 [file JCMM-24-5565-s005.doc]

| **Cell Lines** | **MTAP status** | **MTA content (pmol/milion cells) (mean ± SD)** |
| --- | --- | --- |
| LP-9 | negative | 28,5 ± 3,5 |
| HMC-NEO | negative | 73,8 ± 12,2 |
| REN | negative | 162,9 ± 39,3 |
| MPP 89 | negative | 144,9 ± 62,4 |
| IST-Mes1 | positive | 290,3 ± 130,5 |
| IST-Mes2 | positive | 364,0 ± 29,5 |
| MMB-1 | positive | 109,2 ± 10,6 |
| NCI-H2452 | positive | 254,2 ± 88,2 |

Table S3. MTA content (pmol/milion cells) values expressed as mean ± standard deviation (SD).
